# Calorie-restricted oat diet is associated with zonulin and short-chain fatty acid response in metabolic syndrome: a randomized controlled trial

**DOI:** 10.1080/19490976.2026.2662687

**Published:** 2026-04-23

**Authors:** Linda Klümpen, Aakash Mantri, Anna Donkers, Waldemar Seel, Birgit Stoffel-Wagner, Martin Coenen, Matthias Schmid, Leonie Weinhold, Fabian Grein, Patrick Newels, Janis Bedarf, Ullrich Wüllner, Peter Stehle, Marie-Christine Simon

**Affiliations:** a Institute of Nutrition and Food Science, Nutrition and Microbiota, University of Bonn, Bonn, Germany; b Institute for Genomic Statistics and Bioinformatics, University Hospital Bonn, Bonn, Germany; c Institute of Clinical Chemistry and Clinical Pharmacology, Central Laboratory, University Hospital Bonn, Bonn, Germany; d Institute of Clinical Chemistry and Clinical Pharmacology, Study Center Bonn, Clinical Study Core Unit, University Hospital Bonn, Bonn, Germany; e Institute of Medical Biometry, Informatics and Epidemiology, University Hospital Bonn, Bonn, Germany; f biovis Diagnostik MVZ GmbH, Limburg – Eschhofen, Germany; g Department of Movement Disorders (PSB), Centre of Neurology, University Hospital Bonn, Bonn, Germany; h Institute of Nutrition and Food Science, Nutritional Physiology, University of Bonn, Bonn, Germany; i Computational Microbiome & Brain Health, Fraunhofer Institute for Algorithms and Scientific Computing (SCAI), Sankt Augustin, Germany

**Keywords:** Whole grain, leaky gut, microbial metabolite, multiomics analysis, obesity, human intervention study

## Abstract

Oats are associated with positive effects on gut health, but human studies are largely lacking. Therefore, we investigated the effects of two different oat diets on gut permeability makers in individuals with metabolic syndrome, each in a randomized, controlled parallel design. Participants either consumed 3 × 100 g oatmeal/d for 2 d or an adapted control diet, or they integrated 1 × 80 g oatmeal/d into their habitual diet for 6 weeks or maintained it unchanged. Serum zonulin decreased upon 2-d calorie-restricted oat diet compared to baseline, while plasma butyric acid increased compared to the control (*n* = 27). Zonulin reduction correlated inversely with changes in short-chain fatty acids (SCFAs), particularly valeric and butyric acids, which were associated with shifts in microbial composition. During the 6-week isocaloric oat diet, these parameters remained stable (*n* = 22). Our data suggests that alterations in microbiome and related effects on SCFAs upon a short-term calorie-restricted diet with high-dose oats are contributing factors to changes in gut permeability markers. Thus, an intense oat intake might be a suitable and feasible approach to improve obesity-related intestinal barrier dysfunction in metabolic syndrome.

German Clinical Trials Register: 07/28/2020, identifier: DRKS00022169.

## Introduction

Oats (*Avena sativa*) are a valuable food source known for their multiple beneficial physiological effects due to their unique nutrient composition, including high levels of dietary fiber, especially *β*-glucan.^1^ Meta-analyses and systematic reviews have shown that frequent alimentary oat intake is associated with reduced risks of obesity, cardiovascular disease (CVD), type 2 diabetes mellitus (T2D), and gastrointestinal (GI) disorders.^2^ However, to date, the underlying mechanisms of these health benefits are not fully understood. The oat-induced modulation of the gut microbiota might be one relevant mechanism.^3^ It is well established that the gut microbiota plays a pivotal role in human health. A key mechanism linking the gut microbiota to metabolism is its impact on the intestinal barrier.^4-6^ Impaired gut barrier functions characterized by an increased tight junction (TJ) permeability enable the translocation of commensal intestinal bacteria and/or microbial components, such as lipopolysaccharides (LPS), from the intestinal lumen to the systemic circulation.^7^ This process leads to an activation of immune functions and promotes chronic low-grade inflammation, which is a main characteristic of GI and metabolic disorders, such as inflammatory bowel disease,^8^ obesity,^5^ T2D,^5^ and CVD.^4^ Consequently, dietary interventions modulating the gut microbiota have been proposed as a potential strategy to improve intestinal permeability and thus host health. In this context, short-chain fatty acids (SCFAs), the major microbial end products of dietary fiber fermentation, such as oat *β*-glucan, have been shown to be critical for reinforcing intestinal structures and maintaining intestinal barrier functions. SCFAs, especially butyrate, are metabolic fuels for colonocytes and act as signaling molecules, influencing host physiology locally in the gut and systematically in the periphery.^9-12^ These conclusions, however, were mainly derived from cell and animal studies, while convincing evidence from human studies examining the link between diet, SCFAs, and intestinal permeability is largely lacking.

Therefore, our objective was to investigate the effects of two different oat interventions, a short-term, high-dose oat diet under hypocaloric conditions and a six-week moderate oat diet under isocaloric conditions, on gut permeability markers and inflammatory status in adults with metabolic syndrome (MetS), who are at high risk of impaired gut barrier function.^13^^,^^14^ We hypothesized that oat intake reduces gut permeability and low-grade inflammation compared to the oat-free control diets. In addition, we examined the relationship between changes in gut permeability markers, SCFAs, and gut microbiota composition to reveal potential underlying mechanisms.

## Methods

### Study design

Two different oat dietary interventions, each in a randomized controlled, prospective, longitudinal parallel design, were studied at the Institute of Nutrition and Food Science, University of Bonn, Germany, between September 2020 and July 2022, in accordance with the principles of the Declaration of Helsinki and its subsequent amendments. Approval was granted by the Ethics Committee of the Medical Faculty, University of Bonn (approval number: 212/20). The study was prospectively registered in the German Clinical Trials Register (http://www.drks.de, identifier: DRKS00022169, date: 07/28/2020). Written informed consent was obtained from all participants prior to participation.

Participants of the short-term intervention attended two clinic visits, the first before and the second after the 2-d intervention period. Analogously, participants of the six-week intervention were invited to two clinic visits, before and after the six-week intervention period. After the first visit, participants were randomly assigned to the experimental group or the control group of each study using computer-generated randomization tables in a block format with variable block length.^15^ Blood and fecal samples collected before and after each intervention period were analyzed for markers of gut permeability (serum and fecal zonulin, serum LPS, fecal alpha-1-antitrypsin [A1AT]) and inflammation (high-sensitivity C-reactive protein [hsCRP], interleukin 6 [IL-6], and fecal calprotectin), as well as for plasma SCFA levels. The composition of the gut microbiota was analyzed in fecal samples collected before and after each intervention period using 16S rRNA V3V4 gene sequencing. In addition, anthropometric measurements were performed and the participants completed questionnaires on their habitual diet using a 12-month food frequency questionnaire (FFQ), adherence to the study diets, GI tolerability, and well-being.

### Participants

Men and women aged 45**–**70 y with overweight or obesity (BMI ≥ 27.0 < 40.0 kg/m²) and MetS were recruited at the Institute of Nutrition and Food Science, University of Bonn, Germany, through newspaper advertisements, flyers, and social media. Volunteers who expressed interest in the study were screened through initial telephone pre-screening, followed by a face-to-face meeting (including anthropometric measurements and detailed blood analysis) to confirm eligibility.

MetS was diagnosed based on the global consensus definition of the International Diabetes Federation^16^ as central obesity (defined by a waist circumference ≥94 cm for men and ≥80 cm for women) accompanied by at least two of the following five criteria: (1) elevated blood pressure (≥120 mmHg systolic and/or ≥80 mmHg diastolic),^17^ (2) elevated fasting serum triglycerides (≥150 mg/dL), (3) decreased fasting high-density lipoprotein cholesterol (<40 mg/dL for men and <50 mg/dL for women), (4) elevated fasting plasma glucose (≥100 mg/dL), and/or (5) insulin resistance (HOMA-IR > 2.5) (based on the WHO definition^18^). The latter criterion was added after the initial study registration to give more importance to impaired glucose metabolism as a trait of MetS and to counter recruitment difficulties due to the COVID-19 pandemic. The participants' dietary habits corresponded to a Western dietary pattern^19^ without regular oat intake, defined as less than once weekly at least the last 3 months. The use of proton pump inhibitors, acute inflammatory and infectious diseases, as well as vaccinations during study participation, were defined as exclusion criteria for this explicit research question, in addition to the exclusion criteria listed in the pre-registration data (https://drks.de/search/de/trial/DRKS00022169), as they may be independent confounding factors.^20-22^

### Intervention diets

In the short-term intervention, the two study diets were designed as calorie-restricted (1100–1200 kcal/d), high-fiber interventions with a comparable macronutrient composition (carbohydrates: 67 E%, of which fiber 17%, protein: 15 E%, fat: 17 E%). Participants in the oat group (OG) consumed three oat meals daily instead of their habitual diet. Each meal consisted of 100 g of rolled oats (Demeterhof Schwab GmbH & Co. KG, Windsbach, Germany) boiled in water, providing 10 g of fiber. This dietary treatment was based on a special oat cure developed by the German diabetologist Carl von Noorden at the beginning of the 20th century^23^ and is applied as a complementary therapy for diabetes.^24-26^ Participants in the control group (CG) received three standardized meals adapted to the recommendations of the German Nutrition Society^27^ but without oats, comparable to the calorie restriction and macronutrient composition of the oat diet.

During the six-week intervention period, participants in the oat group (OG^6w^) consumed a daily oat meal consisting of 80 g of rolled oats (Demeterhof Schwab GmbH & Co. KG) and providing 8 g of fiber, while maintaining their habitual Western diet under isocaloric conditions. The amount of oats is based on the health claims.^28^^,^^29^ Participants in the control group (CG^6w^) did not change their habitual Western diet and refrained from eating oats.

All meals, the composition of which was calculated using the computer-based program EBISpro based on the German nutrient database Bundeslebensmittelschlüssel (version 2016; Max Rubner-Institut, Karlsruhe, Germany), were prepared by the participants themselves at home in accordance with a standardized protocol. Further details on the intervention diets are provided in the Supplementary Methods.

Participants' adherence to the study diets was assessed in multiple ways: (1) by the number of (un)emptied returned oat packs, (2) by detailed checklists completed by the participants on each intervention day, and (3) by the plasma concentration of the oat-specific biomarkers avenanthramides (AVAs).^15^^,^^30^^,^^31^

### Assessment of GI tolerability and well-being

The GI tolerability and well-being of the participants were assessed on the first and last intervention day using a standardized questionnaire to record the frequency of specific GI symptoms (abdominal pain, flatulence, diarrhea, and nausea) and well-being (headache, uneasiness, dizziness, exhaustion, concentration disorders, and other complaints), adapted according to Wolever et al.^32^ In brief, the frequency of each symptom was reported on a 4-point scale, with ‘0’ denoting ‘at no time’, ‘1’ denoting ‘once a day’, ‘2’ denoting ‘several times a day’ and ‘3’ denoting ‘all day’. Separate scores for GI tolerability and well-being were calculated as the sum of each individual symptom score, with a range of 0**–**12 (GI tolerability) or 0**–**18 (well-being) representing complete and poor tolerance/well-being, respectively.

During the six-week intervention, GI tolerability and well-being were additionally recorded on a weekly basis. Here, the frequency of each symptom was reported on a 5-point scale, with ‘0’ denoting ‘at no time’, ‘1.5’ denoting ‘once or twice a week’, ‘3.5’ denoting ‘three to four times a week’, ‘5.5’ denoting ‘five to six times a week’, and ‘7’ denoting ‘every day of the week’. Separate scores for GI tolerability and well-being were calculated as the sum of each individual symptom score over the entire intervention period, with a range of 0**–**168 (GI tolerability) or 0**–**252 (well-being) representing complete and poor tolerance/well-being, respectively.

### Anthropometric and blood pressure measurements

Body weight and height were measured with an electronic column scale and stadiometer (Seca GmbH and Co. KG, Hamburg, Germany) to the nearest 100 g and 0.1 cm, respectively. Waist circumference was determined to the nearest 0.1 cm midway between the lowest rib and the iliac crest in duplicate at minimal respiration. Body composition (fat mass and fat-free mass) was measured by air displacement plethysmography using a BOD-POD body composition system (Cosmed, Fridolfing, Germany). Office blood pressure and heart rate were measured twice using a semiautomatic blood pressure measurement monitor (Boso Carat Professional, Bosch + Son GmbH and Co. KG, Juningen, Germany) under standardized conditions according to the European and US guidelines.^33^^,^^34^

### Blood sample processing and routine laboratory analysis

Before and after each intervention period, fasting blood samples were collected in the morning between 8:00 and 10:00 after a 12 h fasting period. To verify the presence of MetS, routine laboratory analyses were performed, including serum triglyceride, high-density lipoprotein cholesterol, and insulin levels, as well as plasma glucose concentrations. All analyses were performed within 4 h of blood collection under standardized conditions using the Roche/Hitachi Cobas c system (Roche Diagnostics, Mannheim, Germany) by a certified medical laboratory (Central Laboratory, Institute of Clinical Chemistry and Clinical Pharmacology, University Hospital Bonn, Germany). Method specifications are available online (www.ukbonn.de/ikckp/zentrallabor/leistungsverzeichnis/). The HOMA index was calculated as follows: HOMA-IR = [insulin (mU/L) × glucose (mg/dL)] ÷ 405. If the HOMA index was >2.5, insulin resistance was assumed.^35^

### Measurement of serum zonulin and lipopolysaccharides (LPS)

To assess gut permeability, serum samples were collected before and after each intervention period to measure zonulin and LPS concentrations. Samples were centrifuged for 15 min at 1700 × g and 8 °C after complete coagulation and supernatants were immediately frozen in cryovials at −80 °C. All samples underwent one freeze–thaw cycle before analysis. Zonulin concentration was measured in duplicate using an enzyme-linked immunosorbent assay (ELISA; antibodies-online GmbH, Aachen, Germany), following the manufacturer's protocol, with an additional dilution step (1:10). LPS concentration was analyzed using an ELISA kit (Cusabio Technology LLC, Houston, TX, USA) following the manufacturer's protocol. Both measurements were performed at the Institute of Nutrition and Food Science, University of Bonn, Germany. The inter- and intra-assay variations were 10% and 10% for zonulin, and 10% and 8% for LPS, respectively.

### Assessment of inflammatory status

To assess the inflammatory status of the participants before and after each intervention period, serum hsCRP levels were measured according to routine laboratory analysis (details see above). In addition, plasma samples were collected to determine IL-6 concentration. EDTA samples were centrifuged for 15 min at 1700 × g and 8 °C, and plasma supernatants were immediately frozen in cryovials at −80 °C. All samples were exposed to one freeze‒thaw cycle before analysis. IL-6 concentration was analyzed in duplicate using an ELISA kit (Bio-Techne Ltd., Abingdon, UK) following the manufacturer's instructions at the Institute of Nutrition and Food Science, University Bonn, Germany. The inter- and intra-assay variations were 7% and 5%, respectively.

### Targeted metabolomics analysis of plasma SCFAs

Plasma acetic acid (C2), propionic acid (C3), isobutyric acid (C4), butyric acid (C4), 2-methylbutyric acid (C5), isovaleric acid (C5), valeric acid (C5), and hexanoic acid (caproic acid, C6) were quantified in a targeted multiplex panel by liquid chromatography with tandem mass spectrometry (LC-MS/MS) using a quantitative lipids platform according to Metabolon Method TAM148 (‘LC-MS/MS Method for the Quantitation of Short Chain Fatty Acid (C2 to C6) in Human Plasma and Serum’; Metabolon Inc., Morrisville, NC, USA) as previously described.^36^ Quality control samples met the acceptance criteria for all analytes at all levels (Supplementary Table S1).

### Measurement of fecal calprotectin, A1AT and zonulin

Fresh stool samples were collected before and after each intervention period just before the clinical visits according to a standardized procedure and immediately stored at −80 °C at the study center. To investigate intestinal inflammation and permeability, fecal concentrations of calprotectin, A1AT, and zonulin were analyzed. Fecal parameter measurements were performed using ELISA kits (K6927, K6750, K5600; Immundiagnostik, Bensheim, Germany) according to the manufacturers' protocols. Calprotectin measurements were performed at the Center of Neurology, University Hospital Bonn, Germany. A1AT and zonulin measurements were performed at Biovis Diagnostik MVZ GmbH, Limburg, Germany. All measurements were performed in duplicate. Inter- and intra-assay variations were 10% and 5% for calprotectin, 11% and 8% for A1AT, and 17% and 6% for zonulin, respectively.

### Gut microbiome analysis

Gut microbiome composition was determined by 16S rRNA gene amplicon sequencing. Details of the DNA extraction, library preparation, and sequencing have been published previously.^15^ In brief, total genomic DNA was extracted from 120 mg of stool using ZR BashingBead lysis tubes (Zymo Research, Freiburg, Germany) and the chemagic DNA Stool kit (Perkin Elmer, Rodgau, Germany) according to the manufacturers' instructions, with a mechanical lysis step using the Precellys 24 Tissue Homogenizer (Bertin Instruments, Frankfurt am Main, Germany) after the addition of the lysis buffer. The V3-V4 region of the 16S rRNA gene was amplified in two PCR steps, and amplicon sequencing was performed on the Illumina MiSeq system. The obtained data were processed using QIIME2 (2021.4), and denoising and quality control were performed using DADA2. Sequences were taxonomically assigned using the SILVA reference database (138.1 SSU Ref NR 99). The median sequencing depth was 65,944 (Q1: 60,952; Q3: 72,751) for the short-term intervention and 33,659 (Q1: 30,057; Q3: 37,779) for the six-week intervention.

### Sample size calculation

A recent study investigated the effects of gastrointestinal nutrition therapy on serum zonulin and IL-6 levels and found large effect sizes for the differences between the nutrition and control groups after the intervention (Cohen's d > 1.6).^37^ Assuming an effect size of Cohen's d = 1.6 for each of the main outcomes and type I error probability *α* = 0.0125 (0.05/4, Bonferroni corrected for multiple testing), 22 participants were estimated to provide 80% power with independent-samples 2-sided *t*-tests.

### Statistical analysis

Statistical analyses were performed using SPSS (version 29.0; IBM Crop., Chicago, IL, USA) and R (version 3.6.2; Boston, MA, USA). Figures were created using R and GraphPad Prism software (version 10.0; GraphPad, Inc.).

Data were expressed as mean ± s.e.m. (baseline characteristics as mean ± s.d.) or median with first and third quartiles (Q1; Q3) for continuous data, depending on the data distribution based on visual inspection of normal quantile‒quantile plots. Categorical variables were expressed as frequencies. Nonnormally distributed data underwent a log10-transformation before statistical analysis. Linear regression models were fitted to evaluate whether the oat diets were superior to the corresponding control diets in terms of intestinal permeability, inflammation, and SCFA response. The models were adjusted for (i) the baseline value and (ii) additionally for sex, and the respective control group was selected as the reference group. Beta estimates of the intervention (*β*) with 95% confidence intervals (95% CI), obtained by bias-corrected and accelerated (BCa) bootstrapping method with 1000 BCa samples, were used to evaluate the magnitude of the effect size. To determine the change over time within each diet group, paired Student's *t*-test or Wilcoxon rank test were performed depending on data distribution. Pairwise correlations were analyzed using Spearman's rank correlation coefficients. For all analyses, the significance level was set at *p* < 0.05. For the main outcomes (zonulin, LPS, hsCRP, and IL-6) family-wise error rate correction was applied via the Bonferroni–Holm method to correct for multiple testing (*p*^*adj.*^ < 0.05).^38^

#### Responder and non-responder analysis

In an exploratory approach, we defined responders and nonresponders within the OG according to the relative change (%∆) of valeric acid (VA), with the cutoff value defined by the upper tertile. Differences between responders (VA-R) and nonresponders (VA-NR) were analyzed analogously to the intervention groups.

#### Gut microbiome and multi-omics analysis

To identify diet-induced changes in microbial composition that distinguish the two diet groups in each intervention (short-term intervention: model 1.1, six-week intervention: model 1.2), a sparse partial least squares-discriminant analysis (sPLS-DA)^39^ was performed using the *mixOmics* R package version 6.22.0.^40^ In addition, an integrative analysis for biomarker discovery using latent components (DIABLO)^41^ was applied to investigate the relationship between changes in microbial composition after the diets and clinical markers (zonulin, LPS, hsCRP, IL-6; fecal zonulin, calprotectin, A1AT) and plasma SCFAs (model 2.1, model 2.2). DIABLO, which extends sparse generalized canonical correlation analysis (sGCCA)^42^ to the sPLS-DA framework, aims to identify coherent patterns between different data sets (such as clinical outcomes, microbiome data, and SCFAs) that change with respect to different phenotypes (study groups).^41^ Moreover, the regression mode of sPLS was applied, using the shift in microbiome composition to explain (‘predict’) the changes in the clinical markers (serum zonulin, LPS, hsCRP, IL-6; fecal zonulin, calprotectin, A1AT) and plasma SCFAs in both oat groups (OG: model 3.1, OG^6w^: model 3.2). Considering the repeated-measures design of both intervention trials, the change in the microbiome data normalized by a centered log ratio (CLR) transformation with an offset of one and the absolute change (preintervention value (baseline) subtracted from the postintervention value) in blood and fecal parameters were used as the input for all models.^43^ Furthermore, an sPLS-DA was performed to determine differences in the initial microbial composition between VA-R and VA-NR based on the outcomes in the OG (model 4). The performance of the sPLS-DA and DIABLO models was evaluated using the area under the curve of the receiver operating characteristic (AUC) and the balanced error rate (BER) derived from internal cross-validation. The results were presented as sample plots (sPLS-DA), correlation plots (sPLS), circos plots (DIABLO), and loadings (all models). Further details of the analysis performed are provided in the Supplementary Methods.

## Results

### Participant characteristics

**Figure 1.**
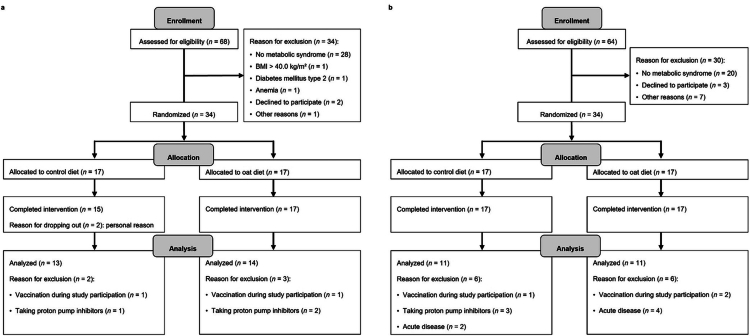
Participants flow diagram for (a) the short-term and (b) the six-week dietary intervention based on the CONSORT flow diagram.

A consort diagram of participants' flow is provided in Figure 1. Participants of the short-term intervention (*n* = 27) were 58.8 ± 7.8 y (mean ± s.d.), 56% females, and had a body mass index (BMI) of 32.1 ± 3.4 kg/m² (Table 1). All participants had central obesity and at least two other MetS traits, including increased blood pressure (100%), dyslipidemia (59%), and impaired glucose metabolism (85%). The participants' habitual diet corresponded to a Western dietary pattern with median (IQR) intakes of 37 (9) E% carbohydrates, 16 (3) E% protein, 44 (5) E% fat, and 19 (10) g fiber. Participants of the six-week intervention (*n* = 22) were 59.1 ± 7.6 y, 55% females, and had a BMI of 31.9 ± 3.1 kg/m² (Table 1). All participants had central obesity and at least two other MetS traits at the screening visit. Thus, at baseline, 100% of the participants had high blood pressure, 45% dyslipidemia, and 73% impaired glucose metabolism. The participants' habitual diet was consistent with a Western dietary pattern, averaging 39 ± 6 E% carbohydrates, 15 ± 2 E% protein, 43 ± 4 E% fat, and 21 ± 6 g fiber. Further details on the baseline characteristics are provided in Supplementary Table S2.

**Table 1.** Baseline characteristics of the participants.^a^

Characteristic	Short-term intervention				Six-week intervention				
	All participants	CG	OG	*p*	All participants	CG^6w^	OG^6w^	*p*	
*n*	27	13	14	–	22	11	11	–	
Sex (female)	15 (88%)	5 (38%)	10 (71%)	0.128	12 (55%)	7 (64%)	5 (45%)	0.670	
Ethnic origin: European	27 (100%)	13 (100%)	14 (100%)	–	22 (100%)	11 (100%)	11 (100%)	–	
Age, years	60.0 (50.0; 66.0)	61.0 (52.5; 66.0)	60.0 (50.0; 64.5)	0.981	59.1 ± 7.6	60.6 ± 6.6	57.7 ± 8.6	0.398	
BMI, kg/m²	31.4 (29.1; 34.1)	31.8 (30.6; 34.2)	30.3 (28.3; 34.5)	0.238	31.9 ± 3.1	31.8 ± 3.7	32.0 ± 2.7	0.843	
Fat mass^a^, %	39.4 ± 6.2	38.7 ± 5.7	40.1 ± 6.9	0.576	42.2 ± 7.6	42.0 ± 8.6	42.3 ± 6.9	0.924	
WC, cm	107.2 ± 9.7	111.7 ± 8.5	103.0 ± 9.2	0.017*	107.6 (104.4; 111.9)	106.0 (100.7; 111.3)	108.8 (107.3; 114.0)	0.193	
BP systolic, mmHg	143.3 ± 16.2	148.8 ± 19.5	138.1 ± 10.6	0.089	137.4 ± 10.0	137.5 ± 11.3	137.3 ± 9.2	0.976	
BP diastolic, mmHg	94.9 ± 11.7	96.9 ± 15.2	93.0 ± 7.4	0.420	88.4 ± 9.9	87.4 ± 10.6	89.5 ± 9.6	0.644	
Triglycerides, mg/dL	157.4 ± 59.6	168.5 ± 74.2	147.1 ± 42.3	0.374	124.0 (94.3; 159.0)	118.0 (77.0; 155.0)	126.0 (96.0; 178.0)	0.274	
HDL-C, mg/dL	49.8 ± 9.8	47.0 ± 6.1	52.4 ± 12.0	0.160	49.0 (41.8; 63.3)	61.0 (45.0; 68.0)	44.0 (41.0; 53.0)	0.092	
Glucose, mg/dL	96.0 (92.0; 108.0)	96.0 (90.5; 109.5)	96.5 (92.0; 108.0)	0.805	96.0 (89.5; 100.8)	93.0 (88.0; 98.0)	99.0 (92.0; 116.0)	0.071	
Insulin, mU/L	18.4 ± 9.5	18.3 ± 6.8	18.5 ± 10.6	0.943	13.3 (8.5; 17.4)	14.0 (8.1; 18.1)	12.6 (9.5; 16.8)	0.877	
HOMA-IR	4.6 ± 2.3	4.5 ± 2.0	4.6 ± 2.6	0.933	3.3 (2.1; 4.0)	3.1 (1.7; 4.1)	3.8 (2.4; 4.0)	0.519	

Note: The significant differences are marked with an asterisk, and the legend explains this as *p < 0.05.

^a^ Data are presented as mean ± s.d. or median (Q1; Q3) for nonnormally distributed data, respectively, or numbers (%). *p < 0.05. ^a^Six-week intervention: *n* = 21.

Abbreviations: BMI, body mass index; BP, blood pressure; CG, control group; CG6w, six-week control group; HDL-C, high-density lipoprotein cholesterol; HOMA-IR, homeostasis model assessment of insulin resistance; OG, oat group; OG6w, six-week oat group; WC, waist circumference.

### Adverse effects and dietary adherence

Overall, the study diets were well tolerated, with generally low symptom scores during both intervention periods (Supplementary Table S3). Participants in the OG reported slightly more adverse effects than those in the CG, particularly on the second intervention day (*p* = 0.011). The most frequently reported diet-related adverse events were flatulence (GI tolerability) and exhaustion (well-being).

Adherence to the study protocol was high in both the short-term (99%) and the six-week intervention (89%).^15^ An expected reduction in body weight and BMI was observed in both diet groups after the 2-d intervention period, with no significant differences between the groups.^15^ During the six-week intervention period, body weight, BMI, and body fat remained stable, confirming that the participants maintained their habitual diet under isoenergetic conditions.^15^

### Short-term, high-dose oat diet decreased zonulin

**Figure 2.**
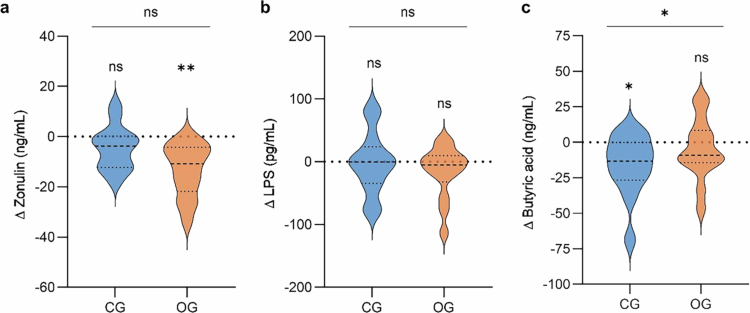
Changes in intestinal permeability markers and butyric acid level following the short-term intervention. Absolute change (shown as ∆) in (a) serum zonulin, (b) serum LPS, and (c) plasma butyric acid concentrations in the CG (blue, *n* = 13) and OG (orange, *n* = 14), presented as violin plots (dashed center line: median, dotted line: upper and lower quartiles). a + b: **p^*adj.*^ < 0.01, ns: *p*^*adj.*^ > 0.05 (Bonferroni Holm), c: *p < 0.05, ns: *p* > 0.05. CG, control group; LPS, lipopolysaccharides; OG, oat group.

Following the 2-d calorie-restricted, high-dose oat diet, serum zonulin level decreased compared to baseline (∆ −13.19 ± 2.79 ng/mL (mean ± s.e.m.), *p*^*adj*^ = 0.002), whereas it remained stable in the CG (∆ −4.88 ± 2.48 ng/mL, *p*^*adj*^ = 0.156) (Figure 2a). However, no significant difference was found between the groups (−7.6 [−16.2, −0.2] ng/mL (*β* estimate OG vs. CG adjusted for baseline [95% CI], *p*^adj^ = 0.216). LPS (Figure 2b), hsCRP, and IL-6 concentrations remain unchanged and did not differ between the two diet groups (Supplementary Table S4). During the six-week intervention period, blood markers of gut permeability and inflammation remained stable with no differences between OG^6w^ and CG^6w^.

Fecal zonulin concentrations declined in the OG; however, this change did not reach statistical significance (∆ −25.88 (−39.37; 13.83) ng/mL, *p =* 0.101). The corresponding change in the CG was smaller (∆ −3.97 (−15.91–32.22) ng/mL, *p =* 0.846), but the OG did not significantly differ compared to CG (−20.8 [−52.4, 11.7] ng/mL, *p* = 0.181). Fecal calprotectin and A1AT also showed no significant within- or between-group differences. Likewise, during the six-week moderate oat consumption, fecal calprotectin concentrations did not differ significantly between OG^6w^ and CG^6w^. Comparable results were also observed after additional adjustment for sex (Supplementary Table S4).

### Different diet-induced SCFA responses

After the 2-d calorie-restricted high-dose oat diet, higher plasma butyric acid levels were observed in the OG compared to the CG (14.8 [5.4, 22.3] ng/mL, *p* = 0.015; Figure 2c). While the butyric acid concentration remained stable in the OG (∆ −4.8 ± 5.7  ng/mL, *p* = 0.514), a reduction was found in the CG (∆ −16.1 ± 5.9 ng/mL, *p* = 0.011). After the six-week intervention period, a different change in hexanoic acid levels was observed between the two groups (11.6 [3.8, 19.3] ng/mL, *p* = 0.040). Hexanoic acid level remained stable in the OG^6w^ (∆ 0.43 ± 5.03, *p* = 0.934), while it decreased in the CG^6w^ (∆ −9.36 ± 4.03 ng/mL, *p* = 0.042). These effects remained significant when additionally adjusted for sex (Supplementary Table S4).

### Diet-induced shifts in gut microbiota composition

**Figure 3.**
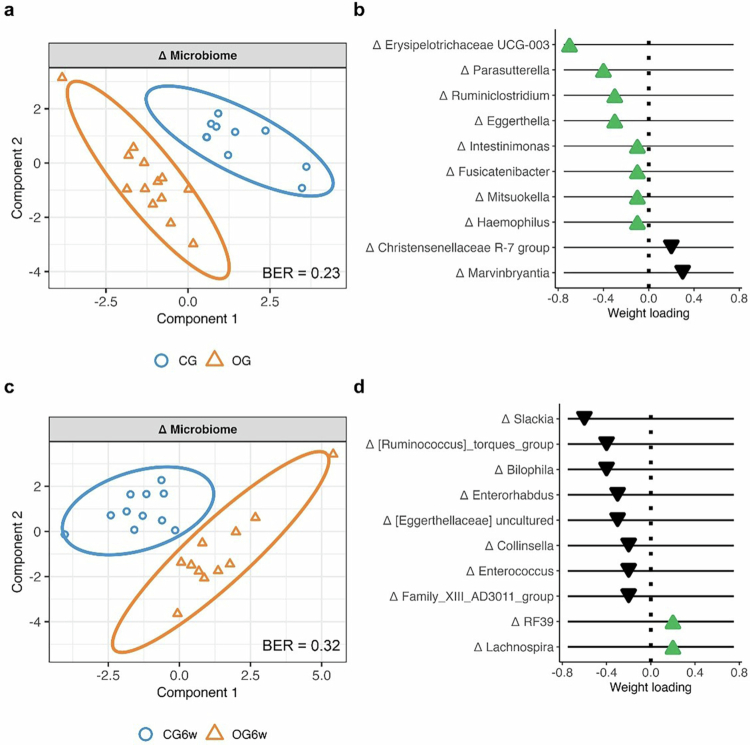
Shifts in microbial composition following the short-term and six-week intervention. (a) Two-component sample plot of the sPLS-DA investigating the diet-induced change in microbial composition at the genus level following the short-term intervention (model 1.1). Data points are shown as blue circles for the CG (*n* = 10) and as orange triangles for the OG (*n* = 13). (b) Weight loadings of the top 10 selected features in component 1 (green or black triangle: increase or decrease in OG vs. CG). (c) Two-component sample plot of the sPLS-DA investigating the diet-induced change in microbial composition at the genus level following the six-week intervention (model 1.2). Data points are shown as blue circles for the CG^6w^ (*n* = 11) and as orange triangles for the OG^6w^ (*n* = 11). (d) Weight loadings of the top 10 selected features in component 1 (green or black triangle: increase or decrease in OG^6w^ vs. CG^6w^). Abbreviations: BER, balanced error rate; CG, control group; CG^6w^, six-week control group; Comp, component; OG, oat group; OG^6w^, six-week oat group.

sPLS-DA revealed changes in microbial composition at the genus level that significantly distinguished the two diet groups of the short-term intervention (model 1.1: BER = 0.23, AUC = 0.78 (*p* = 0.03); Figure 3a). The most important bacteria contributing to the differentiation were Erysipelotrichaceae UCG-003, *Parasutterella*, *Ruminiclostridium*, *Eggerthella,* and *Marvinbryantia*, with the first four showing an increase and the last a decrease in the OG compared to the CG (Figure 3b). In the six-week intervention, a tendency towards a different modulation of the microbial composition at the genus level was observed between the two diet groups (model 1.2: BER = 0.32, AUC = 0.71 (*p* = 0.09); Figure 3c). The bacteria that seemed to be most important for differentiating between OG^6w^ and CG^6w^ were *Slackia*, *Ruminococcus torques* group, *Bilophila*, *Enterorhabdus,* and *Eggerthellaceae* uncultured, all of which decreased in OG^6w^ compared to CG^6w^ (Figure 3d). Further details on all selected bacteria and their weight loadings are provided in Supplementary Table S5.

### Associations between diet-induced changes in clinical markers, SCFAs, and microbial composition

**Figure 4.**
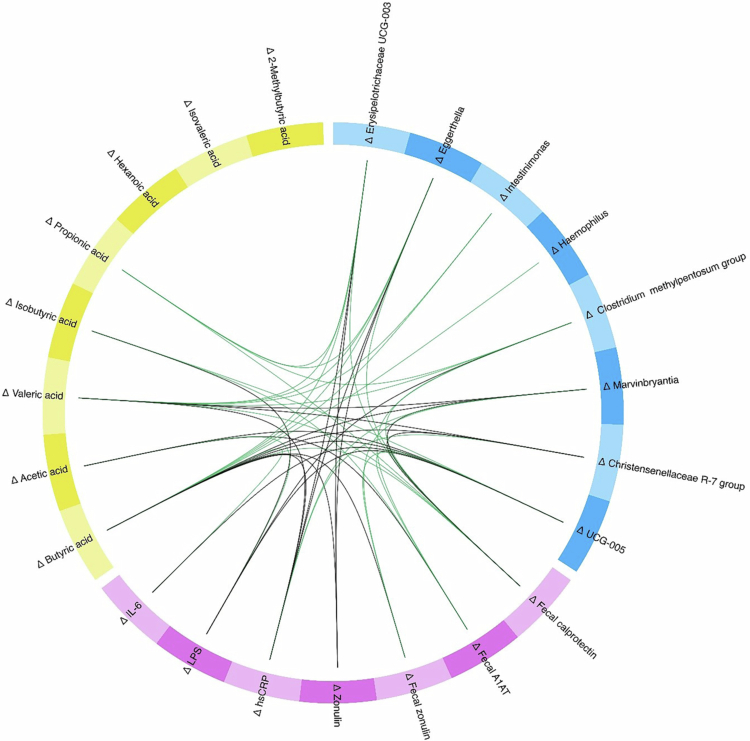
Associations of shifts in microbial composition with changes in clinical markers and SCFAs following the short-term intervention. Circos plot shows positive (green) and negative (black) correlations (cutoff value: *r* = ±0.4) between the selected variables in the three data sets (clinical outcomes (violet), SCFAs (yellow), microbial genera (blue)) along component one and two derived from the DIABLO in the short-term intervention (model 2.1, *n* = 23). Abbreviations: A1AT, alpha-1-antitrypsin; hsCRP, high-sensitivity C-reactive protein; IL-6, interleukin 6; LPS, lipopolysaccharides.

In the short-term intervention, CG and OG were significantly separated in the multivariate DIABLO model (model 2.1: BER = 0.37, AUC = 0.87, *p* = 0.01) and several correlations were revealed between shifts in clinical markers, SCFAs, and specific microbes (Figure 4).

Consistent with sPLS-DA, Erysipelotrichaceae UCG-003 was one of the microbiome factors that contributed most to group differentiation, alongside UCG-005. Regarding clinical markers and SCFAs, changes in zonulin (serum and fecal), A1AT, and changes in butyric and acetic acids were identified as most important (Supplementary Table S6).

Looking more closely at the associations, changes in butyric acid, which showed an increase in OG compared to CG (see above), were inversely associated with changes in zonulin (serum and fecal) and hsCRP levels, as well as with shifts in the abundance of *Marvinbryantia* and Christensenellaceae R-7 group, both associated with an increase in CG. In contrast, positive associations were found with shifts in Erysipelotrichaceae UCG-003, *Eggerthella*, and *Intestinimonas,* showing an increase in OG. Similar patterns were observed for the change in valeric acid (Supplementary Table S6).

In the six-week intervention, CG^6w^ and OG^6w^ were not significantly separated (model 2.2: BER = 0.59, AUC = 0.82, *p* = 0.07); however, DIABLO identified numerous correlations between the datasets (Supplementary Figure 1). Looking more closely at the associations with hexanoic acid, which showed an increase in the OG^6w^compared to the CG^6w^ (see above), an inverse association was found with shifts in LPS and various microbes including *Ruminococcus torques* group, *Slackia,* and *Eggerthellaceae* uncultured (Supplementary Figure 1, Supplementary Table S6). Interestingly, these genera showed a decrease in the OG^6w^ compared to the CG^6w^ (Supplementary Table S5). In contrast, positive associations were observed with shifts in genera such as RF39 and UCG-003, which showed an increase in the OG^6w^ compared to the CG^6w^ (Supplementary Figure 1, Supplementary Table S5).

### Associations between zonulin reduction and SCFA response within diet groups

**Figure 5.**
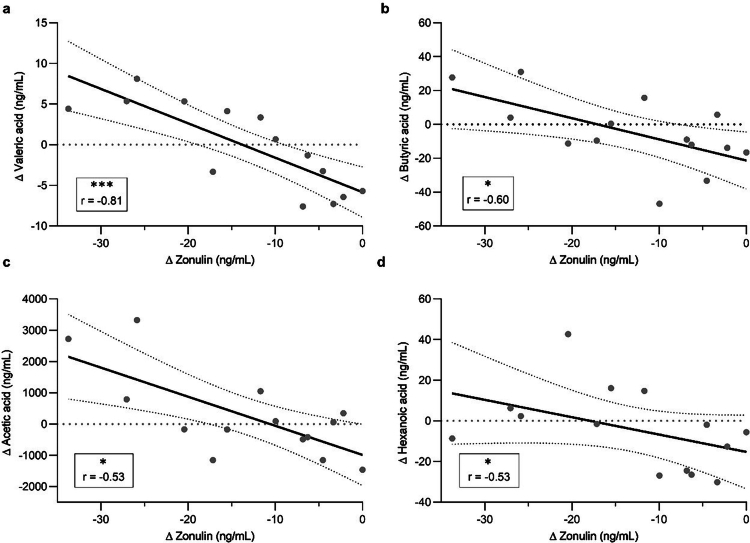
Associations between changes in zonulin and SCFA levels in the OG. Correlation between the absolute change (shown as ∆) in serum zonulin and (a) valeric acid, (b) butyric acid, (c) acetic acid, and (d) hexanoic acid levels. *n* = 14. ***p < 0.001, *p < 0.05.

Looking more closely at the relationships between changes in clinical markers and SCFA levels within each diet group, several inverse correlations were observed between the change in zonulin and shifts in valeric acid (*r* = −0.81, *p* = 4.32 × 10^−4^), butyric acid (*r* = −0.60, *p* = 0.022), acetic acid (*r* = −0.53, *p* = 0.049), and hexanoic acid (*r* = −0.53, *p* = 0.049) in the OG (Figure 5a–d). Positive correlations between the change in zonulin and shifts in butyric acid (*r* = 0.73, *p* = 0.011) and 2-methylbutyric acid (*r* = 0.65, *p* = 0.032) were observed in the OG^6w^. In the CG, a negative correlation was found between the shifts in zonulin and isobutyric acid (*r* = −0.56, *p* = 0.046), while no associations were observed within the CG^6w^ (Supplementary Table S7).

### Associations between microbial shifts and changes in clinical markers and SCFAs within oat groups

Following the 2-d calorie-restricted high-dose oat diet, sPLS regression analysis revealed that shifts in 65 of 135 bacterial genera were associated with changes in clinical markers including serum zonulin, LPS and hsCRP, and all SCFAs (model 3.1; Q2 = −0.23, Supplementary Figure 2). Shifts in bacterial genera associated with a reduction in zonulin were simultaneously associated with an increase in SCFA levels, especially acetic, butyric, and valeric acid. In contrast, shifts in bacterial genera associated with an increase in zonulin were associated with a decrease in these SCFAs.

Following the six-week isocaloric moderate oat diet, sPLS regression analysis revealed that shifts in 7 of 146 bacterial genera were associated with changes in fecal calprotectin, serum hsCRP and plasma 2-MBA, acetic acid, hexanoic acid, and isovaleric acid (model 3.2; Q2 = −0.42, Supplementary Figure 3). Shifts in bacterial genera negatively associated with calprotectin and hsCRP were associated with an increase in SCFA levels, except for acetic acid. In contrast, shifts in bacterial genera positively associated with calprotectin and hsCRP were associated with a decrease in SCFA levels, except for acetic acid.

Details on all identified correlations and the weight loadings of each selected variable in both models are provided in Supplementary Table S7.

### Change in valeric acid associated with zonulin and SCFA response in OG

Because of the negative correlation between the changes in zonulin and valeric acid in the OG (see above), we performed an exploratory analysis investigating differences between VA-R and VA-NR (see Methods). According to the upper tertile of the relative change in valeric acid (%∆ > 6.5%), 6 subjects were identified as VA-R, while 8 were classified as VA-NR.

**Figure 6.**
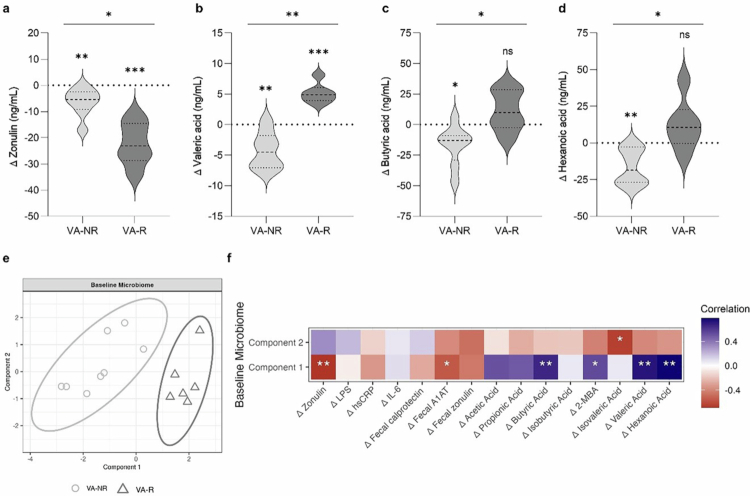
Zonulin and SCFA response in the OG linked to shifts in valeric acid and baseline gut microbiota composition. Absolute change (shown as ∆) in (a) serum zonulin, (b) valeric acid, (c) butyric acid, and (d) hexanoic acid in VA-NR (light gray, *n* = 8) and VA-R (dark gray, *n* = 6), shown as violin plots (dashed center line: median, dotted line: upper and lower quartiles). (e) Two-component sample plot of the sPLS-DA identifying a microbial signature at the genus level that distinguished VA-R (light gray circles, *n* = 8) and VA-NR (dark gray triangles, *n* = 6) at baseline (model 4). (f) Correlations between the baseline microbiome based on the components obtained from the sPLS-DA (model 4) and the changes in clinical markers and SCFAs. ***p < 0.001, **p < 0.01, *p < 0.05, ns *p* > 0.05. Abbreviations: A1AT, alpha-1-antitrypsin; VA-R, valeric acid responders; VA-NR, valeric acid nonresponders; 2-MBA, 2-methylbutyric acid.

As expected, VA-R showed a considerably greater reduction in serum zonulin levels (−17.0 [−28.0, −4.8] ng/mL, *p* = 0.024; Figure 6a) and an increase in valeric acid concentration (9.2 [6.5, 11.7] ng/mL, *p* = 0.002; Figure 6b) compared to VA-NR. Moreover, an increase in butyric acid (20.7 [6.4, 33.7] ng/mL, *p* = 0.031; Figure 6c) and hexanoic acid levels (28.56 [15.1, 37.7] ng/mL, *p* = 0.020; Figure 6d) was observed in the VA-R Compared to VA-NR, primarily driven by SCFA reductions in the VA-NR. These effects persisted when additionally adjusted for sex, apart from hexanoic acid (Supplementary Table S8). In addition, an increase in acetic acid (1,526.7 [357.1, 2445.8] ng/ml, *p* = 0.045) and propionic acid levels (44.0 [24.5, 60.5], *p* = 0.009) was found in the VA-R compared to VA-NR when sex was included in the analysis as a covariate (Supplementary Table S8).

### Baseline microbiome linked to oat-induced zonulin and SCFA response

Comparison of the baseline characteristics between VA-R and VA-NR showed a higher diastolic blood pressure in the VA-R than VA-NR (−8.54 [−15.81, −1.27] mmHg (mean differences [95% CI], *p* = 0.025) and a median difference of −1689 ng/mL for plasma acetic acid (*p* = 0.043) (Supplementary Table S8). Moreover, differences in the sex distribution were observed (*p* = 0.085), as all the VA-R were female, whereas the VA-NR group comprised an equal number of female and male persons.

According to the sPLS-DA, taking into account the unequal sex distribution, a microbial signature was identified that clearly distinguishes VA-R from VA-NR at baseline (model 4: BER = 0.23, AUC = 0.83 (*p* = 0.04); Figure 6e). The most important bacteria for differentiation were *Tyzerella*, Lachnospiraceae NK4B4 group, *Negativibacillus*, *Bacteroides pectinophilus* group, and *Eubacterium eligens* group (top five of ten genera from component one) as well as Clostridia UCG-014 (genus from component two) (Supplementary Table S8). While *Tyzerella*, *Negativibacillus*, *B. pectinophilus* group, and Clostridia UCG-014 were more abundant in the VA-R than VA-NR, Lachnospiraceae NK4B4 group and *Eubacterium eligens* group were less abundant.

Spearman correlation analysis revealed positive associations between component one obtained from the sPLS-DA model and changes in hexanoic acid (*r* = 0.78, *p* = 0.001), valeric acid (*r* = 0.74, *p* = 0.002), and butyric acid (*r* = 0.66, *p* = 0.01) and negative associations with the change in serum zonulin (*r* = −0.69, *p* = 0.008) and fecal A1AT (*r* = −0.56, *p* = 0.046) (Figure 6f). In addition, a negative association was observed between component two obtained from the sPLS-DA model and the change in isovaleric acid (*r* = −0.64, *p* = 0.01).

## Discussion

In the present work, we investigated the effects of two different oat interventions, a short-term, calorie-restricted, high-dose oat diet and a six-week, isocaloric, moderate oat diet, on gut permeability markers and inflammatory status in individuals with MetS in a randomized controlled, prospective, longitudinal parallel design. The high-dose oat diet reduced serum zonulin compared to baseline within 2 d (−31%), alongside a shift in the gut microbiome compared to control. In contrast, caloric restriction alone resulted in smaller, nonsignificant changes in zonulin. Thus, within-group analyses indicated intervention-specific responses, although the high-dose oat diet did not significantly alter markers of gut permeability and inflammation compared to control. However, plasma butyrate was different between groups after the hypocaloric diets. While butyrate declined following the control diet, it did not do so after the high-dose oat diet. Interestingly, changes in circulating zonulin correlated inversely with changes in plasma SCFAs, particularly butyric and valeric acids, which were especially evident in the OG. After the six-week moderate oat intake, gut permeability and inflammatory markers and gut microbiota composition remained stable and only mild effects on the SCFA levels were observed. This suggests that a calorie-restricted high-fiber diet with oats might have the potential to alter systemic gut permeability markers in individuals with MetS, who are at risk for intestinal barrier dysfunction.^13^^,^^14^

Given the increasing prevalence of noncommunicable diseases associated with an impaired gut barrier,^7^^,^^44^ identifying cost-effective and easy-to-implement strategies to restore intestinal barrier function is crucial. Dietary approaches are particularly promising as they can have a wide range of applications, regardless of the metabolic state, without severe side effects as demonstrated in our study. In recent decades, a growing body of evidence from *in vitro* and animal studies has shown that dietary fibers such as oat fiber are able to improve intestinal permeability.^45-48^ In this context, SCFAs produced by the microbial fermentation of dietary fibers are ascribed a key role as they are essential for maintaining and supporting the intestinal barrier function.^9^ Especially, butyric acid has been shown to exert positive effects on the gut barrier function,^10^^,^^49^ supporting our observed inverse association between the change in zonulin and butyric acid levels in the short-term, high-dose oat diet. In addition, valeric acid has been linked to protective effects on the GI tract^50^^,^^51^ and is considered a potential therapeutic agent as shown *in vitro.*^52^ In accordance with these findings, we observed an inverse correlation between the change in valeric acid and serum zonulin levels in the short-term oat intervention, underscoring the need for further investigations of a potential barrier-stabilizing effect of valeric acid. Molecular mechanisms by which SCFAs affect gut permeability include histone deacetylation^10^^,^^52^ and the modification of the MAPK pathway.^53^ However, human trials investigating the relationship between diet, SCFAs, and gut permeability are largely lacking and it should be noted that SCFA levels show considerable inter- and intraindividual variability, especially in blood. One RCT investigating the connection between the Mediterranean diet, fecal SCFAs, and intestinal permeability identified butyric and propionic acids as key mediators linking fiber intake to changes in permeability biomarkers,^54^ which is in line with our findings measured in blood.

In addition, we observed a modulation of the microbial composition, mainly characterized by a higher abundance of genera of the phylum Bacillota (synonym Firmicutes) in the OG compared to the CG. Bacillota represents the primary butyrate-producing bacterial phylum in the human colon,^55^ and previous studies reported an oat-induced increase in Bacillota abundance^56^ as well as a positive association between Bacillota and *β*-glucan fermentation.^57^ At the genus level, an increase in Erysipelotrichaceae UCG-003 and *Intestinimonas*, both of which are associated with an increase in butyric acid, was observed in the OG compared to the CG. Moreover, Erysipelotrichaceae UCG-003 has been associated with healthy aging, potentially by strengthening the intestinal barrier through SCFA production.^58^ *Intestinimonas* is recognized as a butyrate-producing species due to its ability to metabolize sugars and lysine into butyric acid.^59^ This is particularly interesting, as oats contain relatively high levels of lysine,^60^ and the lysine-to-butyrate pathway is estimated to be the second dominant butyrogenic pathway in the intestine, suggesting a contribution of *Intestinimonas* to intestinal butyrate production.^61^^,^^62^ Thus, distinct dietary components like *β*-glucan and amino acids might explain different modulation of the microbiome and butyric acid responses.

Apart from changes in the gut microbiome, the baseline microbiome also appears to play a role in the SCFA and zonulin response to dietary modifications, as a distinct microbial signature was identified between VA-R and VA-NR in the OG. Especially the class Clostridia, which is attributed a specific role in colonocyte metabolism through the production of SCFAs,^63^ seems to be of relevance here. However, given the explorative nature of this analysis and the small sample size, these findings require validation through a hypothesis-driven study with adequate power.

Since caloric restriction also affects gut permeability,^64^ it is worth mentioning that both diets of the short-term intervention were hypocaloric. In line with this, we also observed a trend towards reduced zonulin levels in the CG. Possible underlying mechanisms are a reduction in body weight,^65^ as observed in both diet groups,^15^ and an induction of autophagy,^66^ which is involved in numerous aspects of intestinal physiology,^67^ including the maintenance of the gut barrier integrity by regulating TJ proteins.^68^^,^^69^ Interestingly, a link between butyric acid and autophagy has also been discussed.^70^ In addition, calorie restriction and related weight loss have been shown to influence the composition and function of the gut microbiome, which in turn affects the SCFA concentrations.^71-73^ We hypothesize that the reduction in butyric acid concentration observed in the CG might be related to calorie restriction. While calorie restriction itself might exert beneficial effects on intestinal barrier function,^64^ concurrent decreases in butyrate could have limited the effect in our study. This may have been compensated for by the high-dose oat diet through gut microbiota remodeling, suggesting that the specific prebiotic properties of oats^3^ might have contributed to the reductions in zonulin by maintaining the SCFA concentration stable despite calorie restriction. Together, although the causality of this hypothesis needs to be further confirmed, these observations suggest that the reduction in zonulin in the oat group might reflect the combined presence of calorie restriction and preserved SCFA levels.

Following this hypothesis, we speculate that the stable zonulin and SCFA concentrations after the six-week oat intervention might be explained by the isocaloric design and the lower oat content of the diet, keeping microbial composition almost stable. The fundamentally different nature of the two dietary protocols may also explain the limited overlap in bacteria observed after the short-term and six-week oat interventions, respectively. The differences in intensity of intervention, including deviations from the habitual diet and accompanying dietary factors, are expected to influence the microbiota composition in distinct ways. Thus, a single oatmeal integrated into the habitual Western diet under isocaloric conditions may not be sufficient^74^ to improve gut permeability in individuals at risk of gut barrier dysfunctions. Furthermore, we found a high individual variation in clinical markers at baseline. Interindividual heterogeneity in zonulin concentrations are well-documented, likely reflecting differences in gut microbiota composition, dietary habits, and metabolic status,^75^^,^^76^ which may obscure potential treatment-associated changes. In addition, the modest effects of the six-week moderate oat intervention may be further masked by interindividual differences in the response to oats based on the gut microbiome.^77^

Our study has multiple strengths, including the assessment of the impact of a short-term, high-dose and a six-week moderate oat intake on various clinical outcomes each in a randomized, controlled parallel design with in-depth characterized study groups including males and females; the rigorous monitoring of the respective diets; and the inclusion of three different datasets (clinical outcomes including serum and fecal markers, SCFAs, and gut microbiota) providing a detailed understanding of the intervention effects. However, it is important to acknowledge that the commercially available ELISA kits measure the zonulin family of peptides and not specifically zonulin (prehaptoglobin-2).^78^ This cross-reactivity is an acknowledged limitation of commercially available zonulin assays and may affect the specificity of our findings. Consequently, the observed changes in serum zonulin family peptides should be interpreted as an indirect marker of intestinal permeability rather than a definitive measure of tight junction regulation. Despite this limitation, circulating zonulin levels have been shown to positively correlate with the lactulose/mannitol ratio, a robust indicator of intestinal permeability.^79^^,^^80^ Thus, it is plausible that the reduction in serum zonulin observed following the short-term intervention, although reflecting a systemic rather than a localized marker, is associated with a decrease in gut permeability. Changes in zonulin levels following dietary interventions may therefore still provide valuable insights into gut barrier-related effects.

The sample size was relatively small. This may limit the reliability and generalizability of findings, particularly for outcomes with high interindividual variability or for subgroup analyzes such as the exploratory responder analysis. Therefore, further trials that also ensure adequate statistical power and extent our results are needed. In particular, a multicenter RCT with a well-calculated sample size, including sex-specific differences, would provide stronger evidence and reduce potential center-specific bias, thereby improving external validity across different clinical populations. Moreover, investigations combining localized assessments of intestinal permeability, such as the lactulose-mannitol test or tissue biopsies, with systemic biomarkers as well as functional profiling of the gut microbiome through shotgun metagenomics, are warranted to confirm causal relationships and provide a more comprehensive picture of diet-specific effects along the gastrointestinal tract and subsequent systemic effects.

In conclusion, the observed changes in zonulin and SCFA levels indicate the physiological relevance of a calorie-restricted high-fiber diet with oats, potentially decreasing gut permeability in individuals with MetS. The combination of calorie restriction and oat-specific effects on the gut microbiome and SCFA levels might be considered as a relevant underlying mechanism. From this perspective, a short-term, hypocaloric, high-dose oat diet might be a tolerable and readily implementable approach to alleviate obesity-related increased gut permeability. Further studies are needed to confirm our results and elucidate the potential to improve metabolism by strengthening the gut barrier.

## Supplementary Material

Supplementary Tables.zip Supplementary Tables.zip

Supplementary Material.pdf Supplementary Material.pdf

CONSORT 2010 Checklist.pdf CONSORT 2010 Checklist.pdf

## Data Availability

The raw sequencing data have been deposited in the open repository *Zenodo* under the digital object identifier: 10.5281/zenodo.16271189. These data and the deidentified personal data that support the findings of the current manuscript are not openly available due to reasons of sensitivity and are available from the corresponding author upon reasonable request. Access to the data and additional related documents can be obtained by contacting the corresponding author (Jun. Prof. Dr. Marie-Christine Simon, e-mail address: mcsimon@uni-bonn.de) and signing a data-sharing agreement.

## Abbreviations

A1AT: alpha-1-antitrypsin
AUC: area under the curve of the receiver operating characteristic
BCa: bias-corrected and accelerated bootstrapping method
BER: balanced error rate
BMI: body mass index
CG: control group
CG^6w^: six-week control group
CLR: centered log ratio
DIABLO: Data Integration Analysis for Biomarker discovery using Latent cOmponents
GI: gastrointestinal
HsCRP: high-sensitivity C-reactive protein
IL-6: interleukin 6
LC-MS/MS: liquid chromatography with tandem mass spectrometry
LPS: lipopolysaccharide
MetS: metabolic syndrome
OG: oat group
OG^6w^: six-week oat group
SCFA: short-chain fatty acid
Sgcca: sparse generalized canonical correlation analysis
sPLS-DA: sparse partial least squares-discriminant analysis
TJ: tight junction
V: visit
VA-NR: valeric acid non-responders
VA-R: valeric acid responders
