# Supplementary material for: Calorie-restricted oat diet is associated with zonulin and short-chain fatty acid response in metabolic syndrome: a randomized controlled trial
Source: Gut Microbes. 2026 Apr 23;18(1):2662687. doi: 10.1080/19490976.2026.2662687 (PMC13114110; doi:10.1080/19490976.2026.2662687)
Supplement: Supplementary Material.pdf [file KGMI_A_2662687_SM7529.pdf]

SUPPLEMENTARY MATERIAL

Reduction of gut permeability induced by calorie-restricted oat diet is associated with short-chain fatty acid response

Linda Klümpen, Aakash Mantri, Anna Donkers, Waldemar Seel, Birgit Stoffel-Wagner, Martin Coenen, Matthias Schmid, Leonie Weinhold, Fabian Grein, Patrick Newlers, Janis Bedarf, Ullrich Wüllner, Peter Stehle, Marie-Christine Simon

Corresponding author: Marie-Christine Simon, Institute of Nutrition and Food Science, Nutrition and Microbiota, University of Bonn, Katzenburgweg 7, 53115 Bonn, Germany; Email: mcsimon@uni-bonn.de

SUPPLEMENTARY METHODS

Intervention diets

To increase adherence to the short-term, high-dose oat diet, participants in the OG were allowed to add small portions of fruit (apples, pears, or berries) and vegetables (spinach or leeks) to the oat meals. The standardized meals of the CG contained the same quantity of fruits and vegetables as in the oat diet. The use of salts, sugars and sweeteners was not permitted. For standardization purposes, all participants were instructed to consume a carbohydrate-rich dinner in the evening before starting the intervention. Within the six-week intervention study, the oat meals were adapted to the daily calorie requirements of each participant individually as measured by indirect calorimetry using Quark-RMR® (Cosmed, Fridolfing, Germany).

Gut microbiome analysis

To identify the diet-induced changes in microbial composition that distinguish the two diet groups in each intervention study (model 1.1: OG vs. CG, *n* = 23; model 1.2: OG^{6w} vs. CG^{6w}, *n* = 22), sPLS-DA[1] was applied. The sPLS-DA models were built using the least absolute shrinkage and selection operator (LASSO)[2] and 10-fold cross-validation repeated 50 times. Participants without post-intervention data were excluded (short-term intervention: *n* = 4). The analysis was performed at genus level with a relative abundance threshold of 0.01% (short-term intervention: *n* = 141 bacterial genera; six-week intervention: *n* = 146 bacterial genera). To investigate the relationship between diet-induced changes in blood parameters and microbial composition, DIABLO[3] was applied (short-term intervention: model 2.1, *n* = 23; six-week

intervention: model 2.2, $n = 22$). The models were built with a design matrix of 0.5, as initial pairwise PLS comparisons between the data sets showed correlations ranging 0.64–0.79 (short-term intervention) and 0.40–0.84 (six-week intervention), and with a 10-fold cross-validation repeated 50 times. To determine the relationship between the different datasets (clinical markers, microbiome data, and plasma SCFAs) within the oat groups, the regression mode of sPLS was applied, using the shift in the microbial composition to explain (predict) the changes in the blood parameters (short-term intervention: model 3.1, $n = 13$; six-week intervention: model 3.2, $n = 11$). The model was built with LASSO[2] and leave-one-out cross-validation. To identify differences in the initial microbial composition at the genus level ($n = 148$ bacterial genera) between responders and non-responders based on the outcomes in the OG (model 4, $n = 14$), a sPLS-DA model was built using LASSO[2] and leave-one-out cross-validation. As all responders were female, the removeBatchEffect function was used from the limma package[4] to remove the confounding effect of sex while preserving the effect of the study group.

References

1. Lê Cao, K.-A., Boitard, S. & Besse, P. Sparse PLS discriminant analysis: biologically relevant feature selection and graphical displays for multiclass problems. *BMC Bioinformatics* **12,** 253 (2011).
2. Tibshirani, R. Regression Shrinkage and Selection Via the Lasso. *J. R. Stat. Soc. Series B Stat. Methodol.* **58,** 267–288 (1996).
3. Singh, A. *et al.* DIABLO: an integrative approach for identifying key molecular drivers from multi-omics assays. *Bioinformatics* **35,** 3055–3062 (2019).
4. Smyth, G. K. limma: Linear Models for Microarray Data. In *Bioinformatics and Computational Biology Solutions Using R and Bioconductor,* edited by R. Gentleman, V. J. Carey, W. Huber, R. A. Irizarry & S. Dudoit (Springer-Verlag, New York, 2005), pp. 397–420.

SUPPLEMENTARY FIGURES

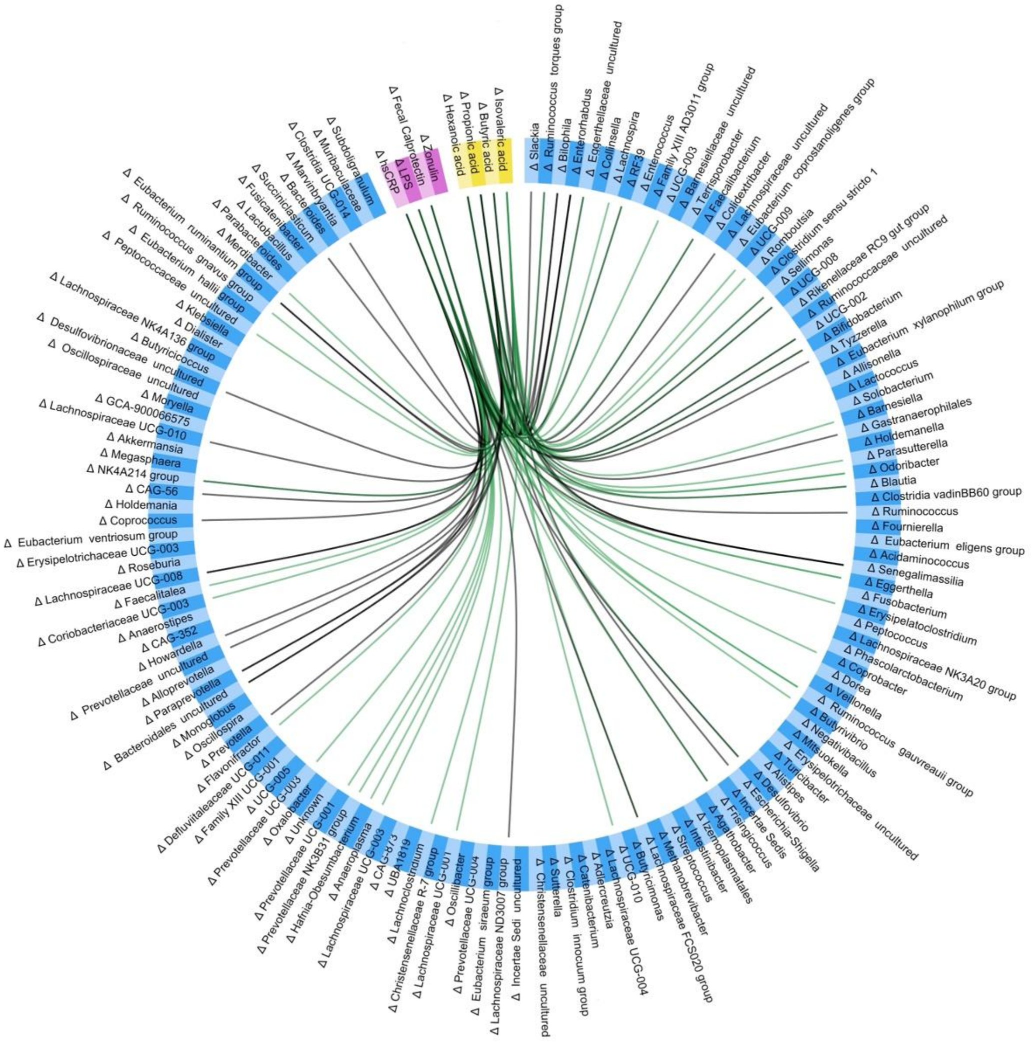

Figure S1. Shifts in microbial composition and associations with changes in clinical markers and SCFAs following the six-week intervention.

Circos plot shows positive (green) and negative (black) correlations (cut-off value: r = ±0.6) between the selected variables in the three data sets (clinical outcomes (violet), SCFAs (yellow), microbial genera (blue)) along component one and two derived from the DIABLO in the six-week intervention (model 2.2, n = 22). Abbreviations: hsCRP, high-sensitivity C-reactive protein; LPS, lipopolysaccharides.

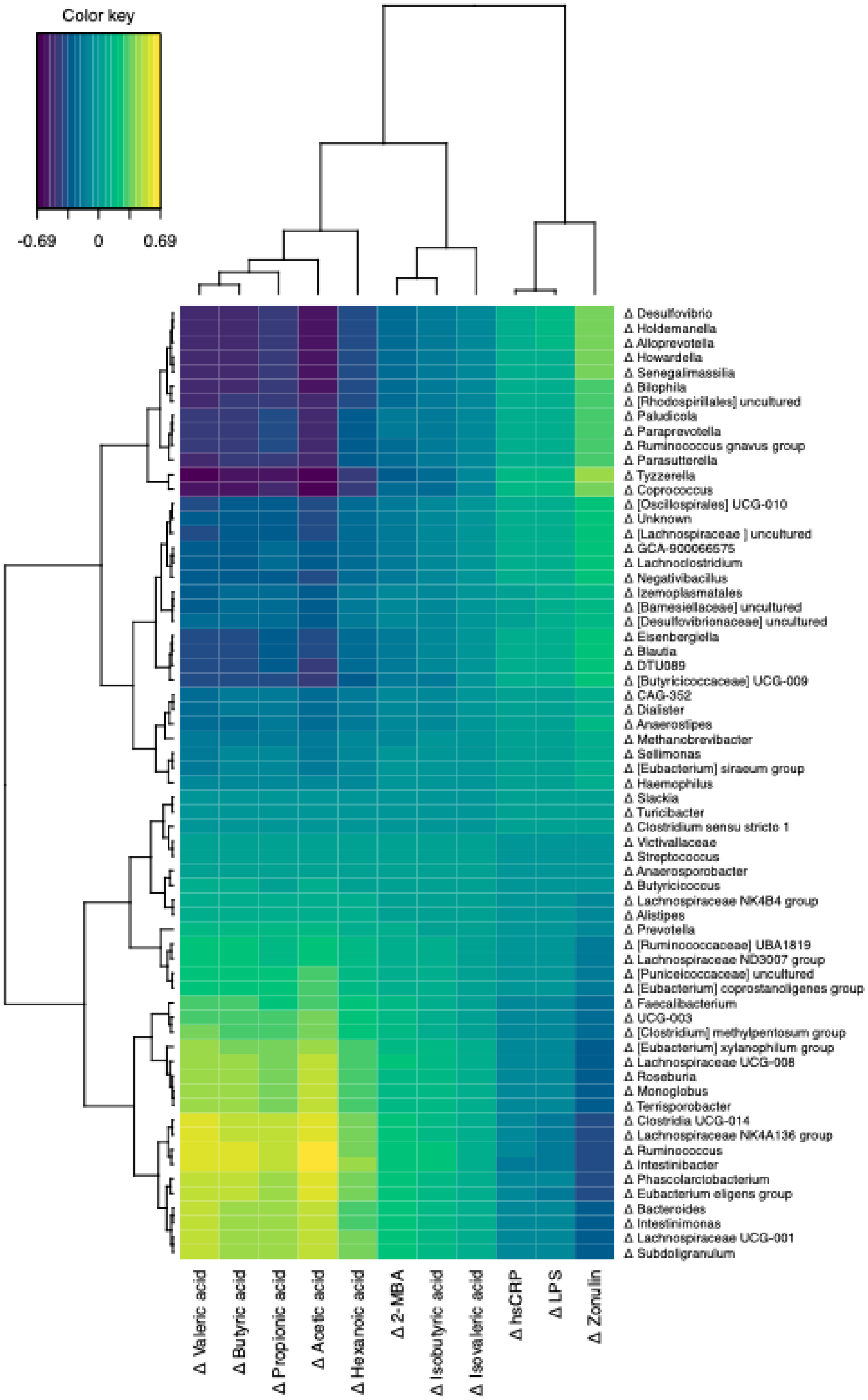

Figure S2: Associations between shifts in microbial composition and changes in clinical markers and SCFA levels.

Figure S2: Associations between shifts in microbial composition and changes in clinical markers and SCFA levels.

Visualization of the sPLS model in regression mode, explaining changes in clinical markers and SCFAs through shifts in gut microbiome composition at genus level following the short-term, high-dose oat diet (model 3.1, OG: n = 13).

Abbreviations: hsCRP, high-sensitivity C-reactive protein; LPS, lipopolysaccharides; 2-MBA, 2-methylbutyric acid.

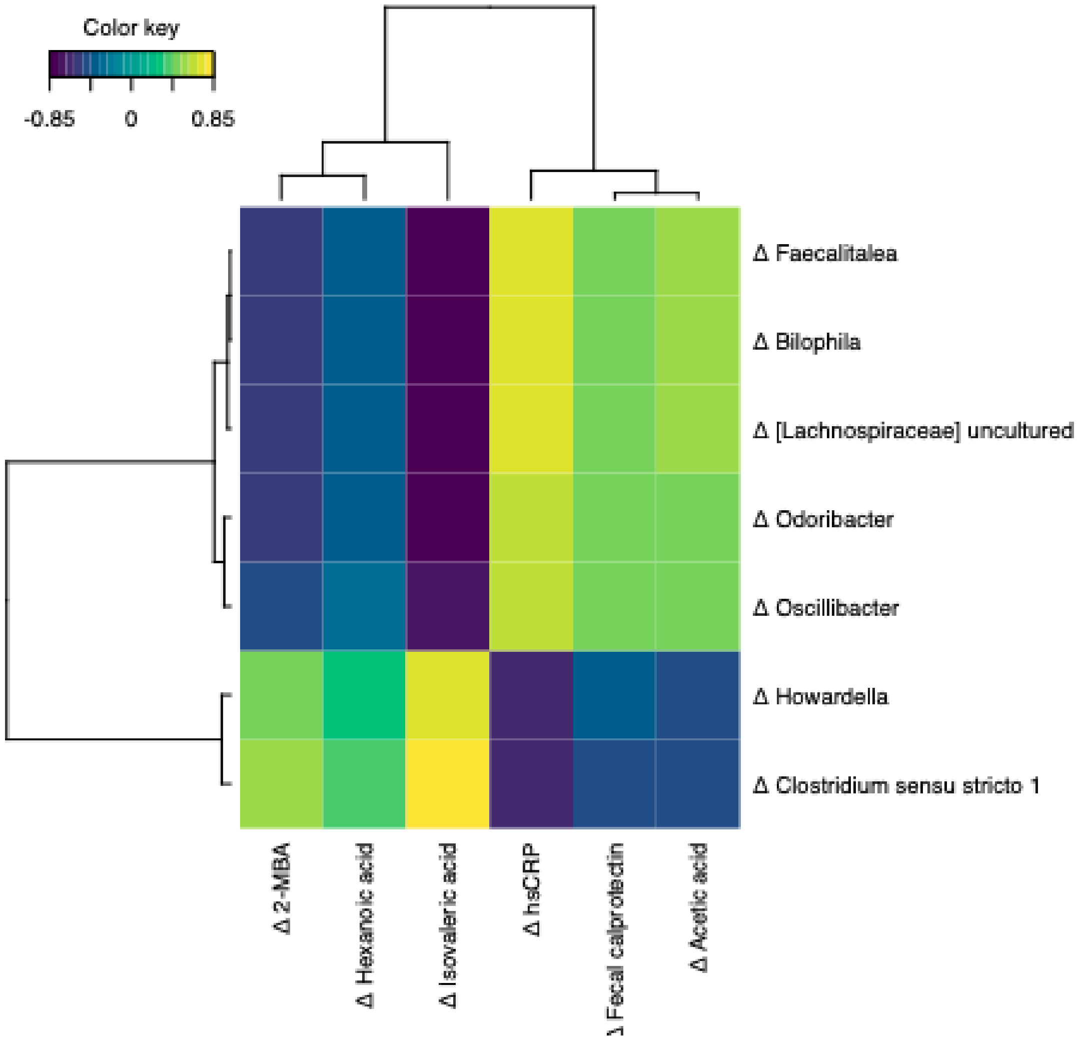

Figure S3: Associations between shifts in microbial composition and changes in clinical markers and SCFA levels.

Visualization of the sPLS model in regression mode, explaining changes in clinical markers and SCFAs through shifts in gut microbiome composition at genus level following the six-week, moderate oat diet (model 3.2, OG6w: n = 11).

Abbreviations: hsCRP, high-sensitivity C-reactive protein; 2-MBA, 2-methylbutyric acid.
